# Heutagogy through Facebook for the Millennial learners

**DOI:** 10.15694/mep.2017.000194

**Published:** 2017-11-02

**Authors:** Prashanti Eachempati, Kiran Kumar KS, Ramnarayan Komattil, Abdul Rashid Hj Ismail

**Affiliations:** 1 Melaka-Manipal Medical College; 2 Melaka-Manipal Medical College

**Keywords:** Heutagogy, Facebook, Social Media, Self Determined Learning

## Abstract

This article was migrated. The article was marked as recommended.

Millennials are the most prevalent generation of medical learners today. These individuals have a unique outlook on education and have different preferences and expectations than their predecessors. The majority of our learners belong to the Millennial Generation, while most faculty belongs to the Baby Boomer or Generation X cohorts. Millennial learners have distinct perspectives on their learning needs that differ with their faculty’s perspectives on teaching and learning. These learning differences may contribute to intergenerational conflict. In order to be successful teachers, it is important to educate ourselves on these generational differences and cater to the needs of the learners. Heutagogy is a self-determined approach of learning, which appears to suit the millennial learners.

Derived from the word “ *Heureskein*” which means to discover, the term heutagogy was coined to describe self-learning, independent of formal teaching. This adds yet another learning theory to the established fields of pedagogy (child learning) and andragogy (adult learning). Heutagogy acknowledges that learner do immensely valuable work for themselves by filling in the gaps of their formal education through discovery and reflection. Heutagogy offers models of learning that are (1) self-determined, (2) peer-led, and (3) non-linear. These characteristics map onto social media applications and the democratization of knowledge and information. Heutagogical approach is an extension of the traditional andragogical and adult learning frameworks through its emphasis on meta learning, or learning how to learn.

This article presents the paradigm shift of educational strategies from pedagogy to andragogy to heutagogy and focuses on the ground principles governing heutagogy. It also describes an innovative case study where principles of heutagogy were applied to train dental students using Facebook.

## Introduction

**Chart 1.**
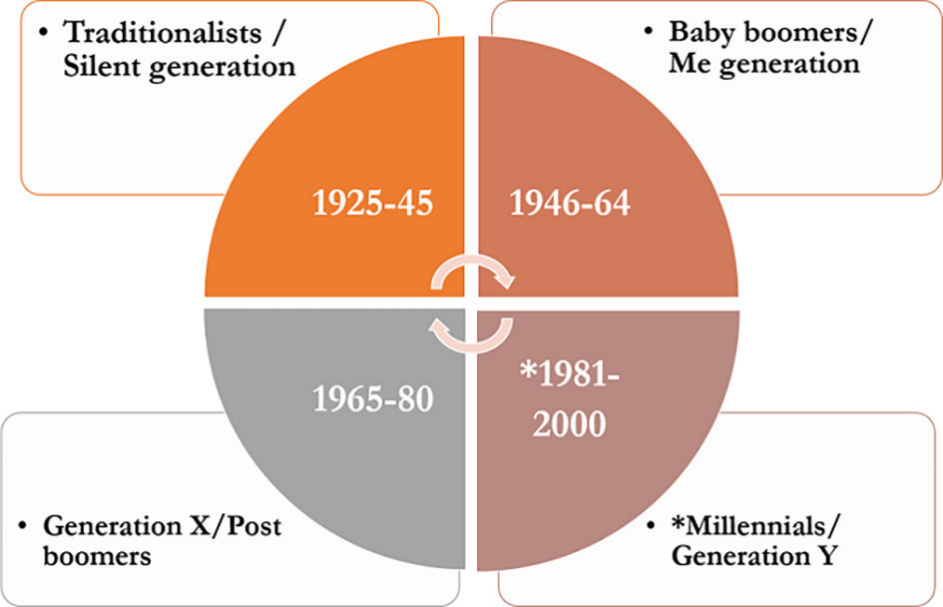
Classification of Generations

Creating competent and capable learners are critical to life in the rapidly changing educational system. Pedagogical, even andragogical educational methods are no longer sufficient in preparing learners for thriving in the workplace, and a more self-determined approach is needed, especially for the millennial learners or the generation Y ( Kuit J A & Fell A 2010; Eckleberry-Hunt J and Tucciarone J 2011). Millennials are the individuals born from 1981 to 2000. This generation of students have shorter attention span and prefer interactive and collaborative learning. They prefer autonomy and are very comfortable with technology. Core workplace values for this generation includes online social media connectedness, teamwork, free expression, creativity, work life balance and use of technology. The major challenge for educators is that majority of our learners belong to the Millennial Generation, while most faculty belongs to the Baby Boomer or Generation X cohorts ( Mohr NM, Moreno-Walton L, Mills AM, Brunett PH & Promes SB 2011) ( Chart 1)

The change in the traits and needs of the learner forces the educational system to adapt to the learner and not vice versa. Heutagogy is a self-determined approach of learning, which suits and caters to the needs of the millennial generation ( Eckleberry-Hunt J & Tucciarone J 2011). In this approach, the learners need to reflect on what is learned and how it is learned and educators teach learners how to teach themselves ( Peter O 2001; Abraham R R & Komattil R 2017).

Practices and principles of heutagogy are rooted in andragogy and has recently resurfaced as a learning approach. Even though its inception dates back to the year 2000, heutagogy has received limited attention from higher education due to the academic resistance to change and a fear of relinquishing the power from instructor to student ( Blaschke L M 2012). However, with the paradigm shift in education towards learner centeredness, heutagogical principles are coming into light. In a heutagogical approach to teaching and learning, learners are highly autonomous and self-determined and emphasis is placed on development of learner capacity and capability with the goal of producing learners who are well-prepared for the complexities of today’s workplace ( Ashton J & Newman L 2006; Bhoryrub J et.al 2010; Hase S & Kenyon C 2000).

## Principles of Heutagogy

### Heutagogy as an extension of andragogy

**Figure 1.**
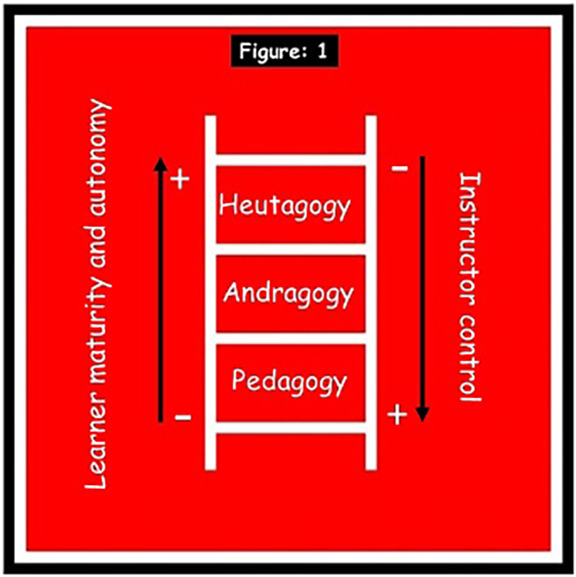
Paradigm shift from pedagogy to heutagogy

Heutagogy meaning “self” in Greek was defined by Hase and Kenyon in 2000 as the study of self-determined learning. Heutagogy is based on a holistic approach to develop learner capabilities, with learning as an active and proactive process, and learners serving as “the major agent in their own learning, which occurs as a result of personal experiences” (Hase S & Kenyon C 2007). Heutagogy is very similar to the andragogical principles and can be said to be an extension of andragogy. As in andragogy, even heutagogy advocates the principle that instructor facilitates learning process by providing guidance and resources. However, unlike in andragogy where the teacher still has control over the learning process, heutagogy fully relinquishes ownership of the learning path and process to the learner, who negotiates learning and determines what will be learned and how it will be learned ( Hase S & Kenyon C 2000; Eberle J 2009). ( Figure 1)

### Competence versus capability

Heutagogical learning environment facilitates development of capable learners and emphasizes on both development of learner competencies as well as development of the learner’s capability and capacity to learn ( Hase J & Kenyon C 2000; Ashton J & Newman L 2006; Bhoryrub J et.al. 2010).

Competency can be understood as proven ability in acquiring knowledge and skills, while capability is characterized by learner confidence in his or her competency and, as a result, the ability “to take appropriate and effective action to formulate and solve problems in both familiar, unfamiliar and changing settings” ( Cairns L 2000).

Capable people exhibit self-efficacy in knowing how to learn and continually reflect on the learning process, can work well with others and be openly communicative. They are creative in applying competencies to new and unfamiliar situations and are positive, adaptable and flexible in their approach. (Gardner A et al. 2007; Hase J & Kenyon C 2000; Kenyon C & Hase J 2010). When learners are competent, they demonstrate acquisition of knowledge and skills; skills can be repeated and knowledge retrieved. When learners are capable, skills and knowledge can be reproduced in unfamiliar situations. Capability is hence the extension of one’s own competence, and without competency, there cannot be capability. ( Snowden M & Halsall, J P 2016).

#### Single loop learning versus double loop learning and reflection:

**Figure 2.**
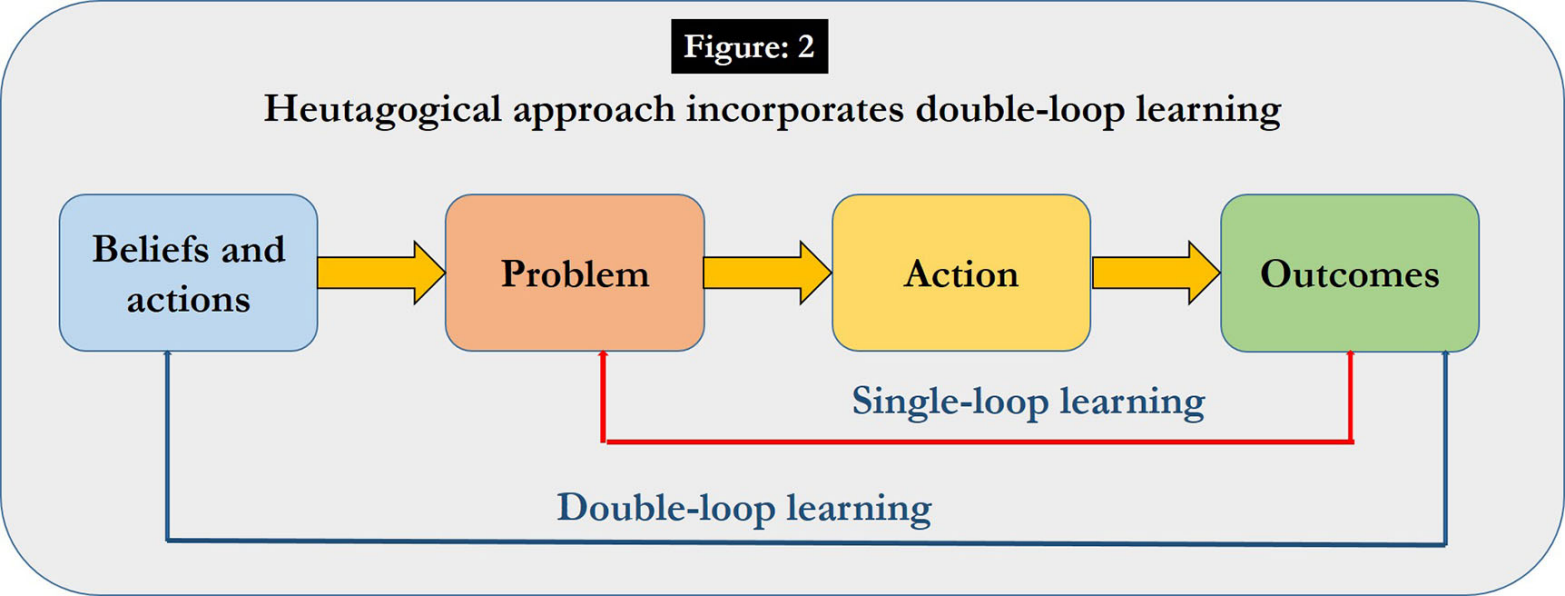
Double loop learning in heutagogical approach

A key concept in heutagogy is that of double-loop learning and self-reflection ( Argyris C & Schön D 1996). In double-loop learning, learners consider problems and resulting action and outcomes, besides reflecting on the problem- solving process and how it influences learner’s own beliefs and actions. Double-loop learning occurs when learners “question and test one’s personal values and assumptions as being central to enhancing learning how to learn” ( Hase S 2009). ( Figure 2)

Another dually important characteristic of heutagogy is that of reflective practice, “a critical learning skill associated with knowing how to learn” ( Hase S 2009). According to Schön D A 1983, reflective practice supports learners in becoming lifelong learners and is based on the principle that “when a practitioner becomes a researcher into his own practice, he engages in a continuing process of self-education”.

### Methods used to apply heutagogy in education

Literature reports application of heutagogical approach to web-based learning ( Blaschke L M 2012), distance learning methods ( Peters, 2001), e-learning modules and blended learning. Social media like mobile learning ( Anderson T 2010; Cochrane T & Bateman R 2010; Wheeler S 2011), visual philosopher ( Hornsby K L & Maki W M 2008) , twitter ( Junco R, Heiberger G, & Loken E 2010), learner-generated content ( Blaschke L M, Porto S & Kurtz G 2010) have also been reported. Social media has been shown to be beneficial in learning and reported to be a suitable medium for carrying out curricular reforms based on heutagogy as connectivity, interaction and information abundance is its strength. Another pertinent feature of social media is that learner can individualize their learning according to their decisions ( Kuit J A & Fell A 2010).

Studies have reported the use of social media platforms like Twitter, visual philosopher etc to apply the principles of heutagogy. However, to the best of our knowledge none have reported the use of Facebook for heutagogy. In our current study we used Facebook to apply the principles of heutagogy in an undergraduate dental setting as it is the most popular social media.

## Facebook for implementing heutagogical principles for undergraduate dental students - A case study at Faculty of Dentistry, Melaka-Manipal Medical College

### Methodology

Application of heutagogical principles for undergraduate dental students has been less explored in literature. In our case study, we used Facebook as a common platform to engage students in discussions and apply heutagogical principles to solve a complex case of cast partial denture.

**Figure 3.**
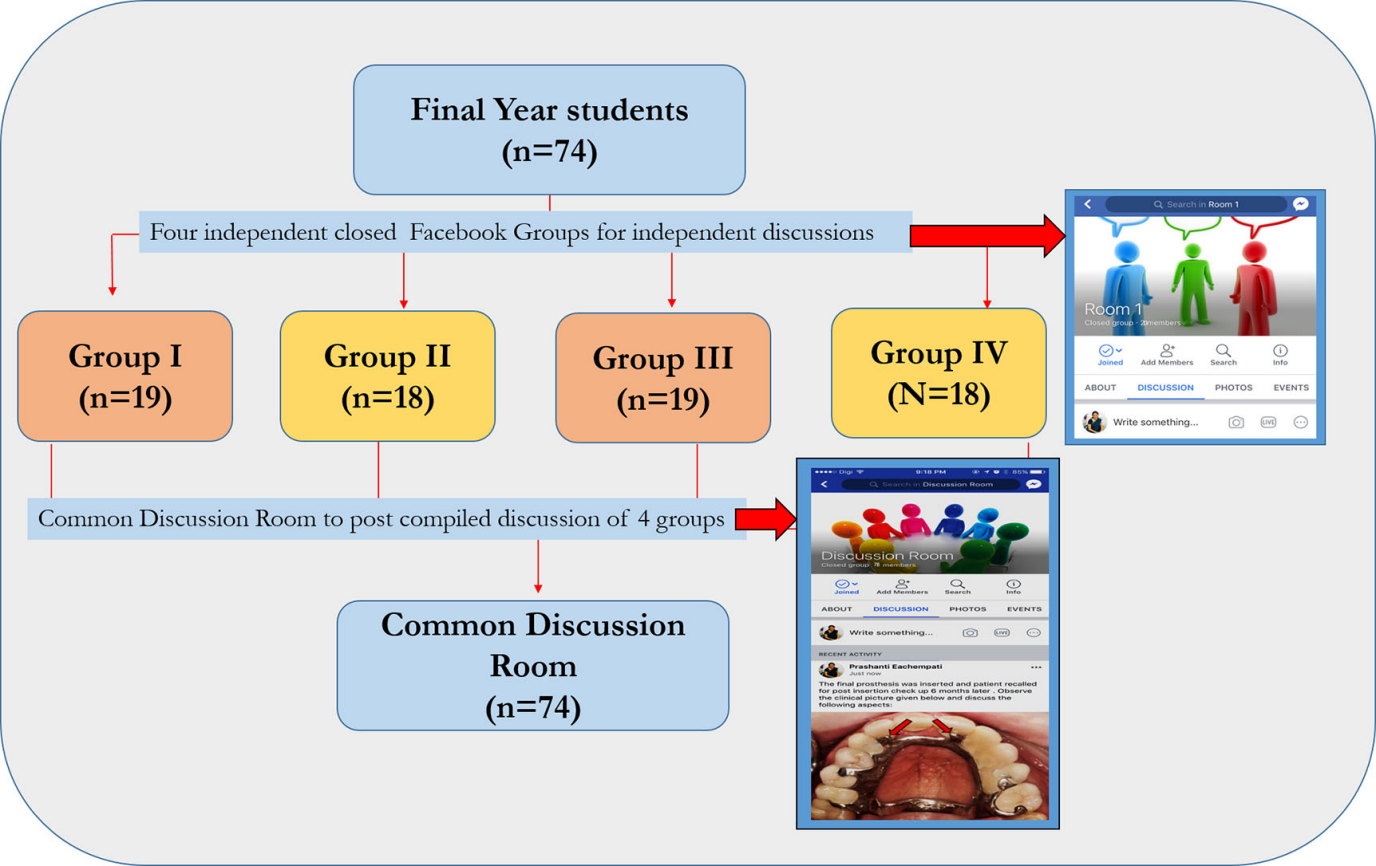
Illustration of groupings in our study

The final year students were divided into four groups. Two facilitators from department of Prosthodontics monitored the group discussions. The students were asked to create four closed Facebook groups for individual discussions (A closed Facebook group will allow only the pre-approved members by the group administrator (facilitator) to see and post comments on the content. Each closed group had 18-19 students and one facilitator enrolled). Apart from four closed groups, a common discussion room was also created with all the 74 students and two facilitators enrolled into it (Common discussion room is also a closed Facebook group, but in this group all the students are enrolled and will be able to see and post their comments) ( Figure 3).

In the first week, a vignette describing a complex partially edentulous case was posted in the common discussion room. All the students read the vignette and started discussion in their respective closed groups. They selected their moderators among their peers, who ensured that all students participated in the discussion. The discussion was asynchronous; hence, the participants had the flexibility to give inputs at the time convenient to them. To keep the discussion on track, the moderators determined the date and time by which all participants were expected to share their inputs. The facilitators observed the discussions in all the closed groups and gave inputs whenever necessary. During week one, the groups formulated learning objectives and topics to be learnt to solve the case. At the end of this week, each group posted their learning objectives in the common discussion room. Students from each group reviewed the inputs of the other groups and discussed to clarify doubts and come to a consensus regarding the learning objectives.

During week two, each group collected pertinent resources and posted in the common discussion room, so that the resources were accessible to all. The facilitators ensured that the resources were from reliable sources. At the end of week two, students in each group listed, the sequence of treatment needed for the case and discussed justifications for their treatment plan in common discussion room. The facilitators gave their comments regarding the final treatment plan and answered queries raised by the students on the common post.

In week three, a second vignette was posted. Different moderators were selected by the groups for facilitating discussions for the second vignette. Once again, the learning content was determined and the students compiled their ideas in the common discussion room as discussed above.

Discussions were moderated in the Facebook groups for four weeks. At the end of the study students reflected on the discussions and their learning which occurred over four weeks and presented the findings in a debriefing session conducted in a classroom. The facilitators gave feedback on all the four-week discussions and identified areas that could be improved. The students noted the inputs, revised their compiled answers (corrective action), and posted in Facebook in the fifth week for final consensus. Student perceptions were collected at the end of fifth week regarding the heutagogical approach via Facebook.

**Ethical Approval:** Ethical approval was obtained after presentation of study protocol to the Research and Ethics Committee, Faculty of Dentistry, Melaka Manipal Medical College, Ref: MMMC/FOD/AR/B4/E C-2016 (26). Only the participants who consented to take part in the study and did not have objection to use Facebook participated in the study.

## Results and Discussion

The perception of students were qualitatively analysed. We used the in-vivo coding using direct language of participants to create codes. Open coding was done using a line-by-line process. After the first cycle of coding, code mapping was done to condense the initial codes into a selected list of categories. Axial coding was done to find the dominant codes and remove redundant codes. These emergent categories were further analysed to discern the emergent themes. On completing this process of thematic analysis five themes were generated.

### Independent learning and autonomy

The use of Facebook to apply heutagogy helped learners individualize their learning according to their decisions ( Kuit J A & Fell A 2010). The independence in formulating the treatment plan according to their understanding boosted their confidence. The potential use of technology gave the students more autonomy, which in turn led to higher cognitive engagement with content and tasks ( Rotgans J I & Schmidt H G 2011).

Citing a few examples of student perceptions:

*“I liked that we could independently solve a complex cast partial case without the lecturers spoon feeding us”*

*“I cannot believe we did everything on our own, right from searching for reading material to formulating the treatment plan. This experience boosted my confidence to solve cases independently”*

*“Asking us to do everything from scratch gave us the feeling of authority. We are more confident now to deal with such cases. This approach is excellent”*

### Flexibility in learning

Students thoroughly enjoyed the flexibility with which they could learn. Asynchronous learning ensured that students could learn at their pace and convenience.

A few student reflections are cited below:

*“I could do it at my pace and my convenience. I loved that best”*

*“No time restriction, no compulsion. I could log in at the time I want and take part in the discussion.”*

### Capability, Teamwork and respect for others

The approach of heutagogy in this case study helped learners develop some of the attributes associated with capability such as knowing how to learn, working well with others, respect for others, creativity, critical thinking, self-efficacy, self-confidence and deliberative dialogue ( Gardner et al. 2008; Walker M 2008). These were clearly reflected during the four week discussions and their feedback.

*“I feel more competent and capable to tackle such cases now”*

*“We worked as a team and solved the case”*

*“I learned to respect other’s views and opinions”*

### Effective discussions and instant feedback

Facebook discussions facilitated interaction between learners and teachers. The added advantage was that the students gave and received instant and constant feedback from their peers as well as teachers on the learning process. In this way, the trait of reflective practice ( Hase S 2009; Sandars J & Hart C S 2015) was fostered which may in turn be helpful to them in their journey towards life-long learning. Through the common discussion platform, there was room for learner directed questions which guided learners in developing the learning content ( Blaschke L M 2014). Few examples are cited below:

*“The discussions helped a lot. I got to know where our thinking went wrong”*

*“The best thing was we were correcting our mistakes by seeking inputs from friends and lecturers even before we completely go wrong”*

*“Discussions in the closed groups followed by the common room discussions enabled us to clarify concepts and gradually evolve with our treatment plan”*

### Facebook as a tool for learning

As discussed earlier, the millennials are technology dependent and connected via social media ( Eckleberry-Hunt J & Tucciarone J 2011). Facebook had the added advantage of capturing learners’ interest, excitement and curiosity and that probably makes it a viable tool for supporting heutagogy. The millennial generation of students enjoyed the fact that their favorite and frequently used social media was now a medium for learning.

Examples of student perceptions for this theme:

*“I am amazed to see that Facebook can be used for learning. I thought it’s only for entertainment”.*

*“The minute I heard we are going to learn via Facebook, I knew it would be fun”*

### Miscellaneous

Some reflections that could not be placed into separate themes, but were pertinent are given below:

“ *Some participated more and some less. This should be carefully monitored*”

*“More photographs accompanying the vignette will help us visualize the case better”*

The debriefing session involving the students to present their reflections, and also to do corrective actions enabled double-loop learning ( Argyris C & Schon D 1978).

*“The de-briefing session gave us a comprehensive view of the four weeks and we understood where we could improve”*

The heutagogical framework we carried out using Facebook aimed at providing more autonomy-supportive learning experiences to millennial learners, thereby increasing their cognitive engagement, their progression to competencies and capabilities. ( Rotgans J I & Schmidt H G 2011).

## Conclusion

The Facebook intervention for applying heutagogical principles for the millennials was an innovative and novel experience. We as facilitators, realized that our role is limited to that of a navigator and learner autonomy is the key to successful implementation of heutagogy. We need to accept that the role of teachers in a heutagogical model would deploy scaffolding in which they support learners’ progression towards competencies and provide opportunities for them to accomplish capabilities. Understanding the learning preferences and needs of the millennials, will avoid intergenerational conflicts and ensure a conducive learning environment.

## Take Home Messages

• In heutagogy, learners serve as the major agent in their own learning, which occurs as a result of personal experience
• Social media can be an effective tool for implementing heutagogy to the millennial generation of students
• The role of the teacher is more of a guide on the side rather than being the sage on the stage

## Notes On Contributors

Prof. Dr. Ramnarayan Komattil: Concept and Idea for the conduct of this study.

Prof. Dr. Abdul Rashid Hj Ismail: Support and guidance during the conduct of the study.

Prof. Dr. Prashanti Eachempati : Principal investigator and primary author for manuscript preparation.

Assoc.Prof. Dr. Kiran Kumar KS: Co- Investigator and co-author for manuscript preparation.
