# Supplementary material for: Heutagogy through Facebook for the Millennial learners
Source: MedEdPublish (2016). 2017 Nov 2;6:194. [Version 1] doi: 10.15694/mep.2017.000194 (PMC10885314; doi:10.15694/mep.2017.000194)
Supplement: Peer review history

# Reviewer report: Reviewer response for version 1

**DOI:** 10.21956/mep.19371.r28356

**Published:** 2018-09-29

**Authors:** Anthony David.M

**Affiliations:** 1 Soho

This review has been migrated. The reviewer awarded 4 stars out of 5 A lucid presentation which was well supported by relevant references, most of which are online. The use of Facebook is near ubiquitous in this generation of professional students. Using it as a self learning tool is a very appropriate technique indeed. As a guide by the side we need to be more empathetic. The issues of misuse of social networking sites and Facebook by medical students and the effects thereof are being a bone of contention in some medical schools in the developing world. Some of them have banned the use of social media on their campuses to avoid their misuse. In this context, such positive use of the same social media needs to be highlighted.

Reviewer Expertise:

NA

No decision status is available

# Reviewer report: Reviewer response for version 1

**DOI:** 10.21956/mep.19371.r28357

**Published:** 2017-11-05

**Authors:** PATRICIA CURY

**Affiliations:** 1 FACERES

This review has been migrated. The reviewer awarded 4 stars out of 5 Dear authors, congratulations for this paper! We can see that a lot of work and effort were done!I agree with Ken Masters´s comments that it is a nice use of Facebook even if the name of the process could be another one.We will try to use the same metholology in our Instituition.

Reviewer Expertise:

NA

No decision status is available

# Reviewer report: Reviewer response for version 1

**DOI:** 10.21956/mep.19371.r28359

**Published:** 2017-11-05

**Authors:** Ken Masters

**Affiliations:** 1 Sultan Qaboos University

This review has been migrated. The reviewer awarded 3 stars out of 5 The authors have tied their practical application of Facebook in medical education into some theoretical aspects of learner-centred education. While there is still much debate about heutagogy, there is certainly much work being done on concepts of learners’ setting their own goals and moving beyond the confines of the LMS in their learning; this paper fits in well with those ideas.The main issue I have with the paper, however, is that it describes a process that falls short of heutagogy. It does not really describe heutagogy; it describes using Facebook groups for online PBL (with a nice use of reflection, as advocated by PBL). This is certainly admirable, and is an important pointer to breaking out of the LMS, but that does not make it heutagogy. For example, if we look closely as Hase and Kenyon’s 2000 paper, we see the following description:“A heutagogical approach recognises the need to be flexible in the learning where the teacher provides resources but the learner designs the actual course he or she might take by negotiating the learning. Thus learners might read around critical issues or questions and determine what is of interest and relevance to them and then negotiate further reading and assessment tasks. With respect to the latter, assessment becomes more of a learning experience rather than a means to measure attainment. As teachers we should concern ourselves with developing the learner’s capability not just embedding discipline based skills and knowledge. We should relinquish any power we deem ourselves to have.”Extremely important here is that the teacher provides resources, but “the learner designs the actual course he or she might take by negotiating the learning”. This is quite different from CBL or PBL in which the case or vignette is created by the teachers and the learners create the Learning Objectives. This provision of the vignette is exactly what is described in this paper, and so is not part of heutagogy.A second crucial part of heutagogy is that the learners determine (or at least negotiate) the assessment. Assessment is not discussed in this paper, so one is left wondering how it was done. If assessment was performed using similar methods (CBL or PBL) that might still fall a little short, but would at least be palatable and close to heutagogy. If, however, assessment followed standard traditional methods (students individually performing assessment tasks (OSCEs, OSPEs, essay, MCQs, MEQs, etc.)), then the researchers will have betrayed their own principles. What good does it do for students to learn new skills of collaborative problem-design and learning objective setting, if that is never examined or assessed? So, for the paper to be complete, the authors should really describe the assessment methods, and how these tied into the heutagogical attempts in the teaching.In addition, some of the other areas of heutagogy, such as learner-created teaching and learning materials were not explored in the research – the students discussed the learning objectives just as they would in f2f PBL.So, while this is certainly a nice use of Facebook in PBL, I would recommend that the authors confine their description of their work to that.I do have one other small criticism: The authors should be careful of too glibly referring to the Millennial Generation and their learning approaches as if they are inherent to such a diverse group of people who have in common only the chronological bracket in which they were born. I would suggest they use the term carefully – it is not necessary for the success of their paper or research, and tying their ideas to it could become problematic.

Reviewer Expertise:

NA

No decision status is available

# Reviewer report: Reviewer response for version 1

**DOI:** 10.21956/mep.19371.r28358

**Published:** 2017-11-02

**Authors:** P Ravi Shankar

**Affiliations:** 1 American International Medical University

This review has been migrated. The reviewer awarded 4 stars out of 5 I enjoyed reading this well written and lucid article. The authors have explained the term ‘heutagogy’ very well. I agree with the authors that the millennials are very comfortable with technology. Social media is widely used and the internet offers a wealth of learning resources. The major challenge may be for teachers. Heutagogy goes beyond andragogy and the teacher relinquishes control of the learning path and process to the learner. My experience of nearly two decades has been that a minority of teachers are extremely reluctant to relinquish control and power. Many believe that students can learn only if they are taught. The situation may however, vary from institution to institution. I have written a number of publications on this topic. The process followed has been described very well by the authors. Reflection is recognized as increasingly important for both learners and teachers. Facebook is widely used and students enjoyed its use for learning as is evident by their comments. The literature mentions the asynchronous nature of social media and the democratization of learning as major advantages. Democratization and the loss of power may be threatening to some teachers and more teacher education may be required. I believe over the generations teachers have become more democratic and less authoritarian. I look forward to more examples and greater use of social media in education from the authors.

Reviewer Expertise:

NA

No decision status is available
